# Three‐step algorithm for biological therapy targeted IgE and IL‐5 in severe asthma

**DOI:** 10.1002/iid3.233

**Published:** 2018-08-07

**Authors:** Keiji Oishi, Kazuto Matsunaga

**Affiliations:** ^1^ Department of Medicine and Clinical Science Graduate School of Medicine Yamaguchi University Ube Yamaguchi Japan; ^2^ Department of Respiratory Medicine and Infectious Disease Graduate School of Medicine Yamaguchi University Ube Yamaguchi Japan

**Keywords:** Atopy, biological therapy, eosinophils, FeNO, severe asthma, steroid trial

## Abstract

**Introduction:**

Recently, several new biological therapies targeted IgE and IL‐5 in severe asthma have been developed and approved. In the last few years, there have been some reports on the therapeutic algorithms for severe asthmatic subjects screened by biomarkers. However, these algorithms have one problem. In atopic/eosinophilic overlapping asthmatic subjects, there is no effective answer to the question: “which is the optimal choice between Anti‐IgE and anti‐IL‐5?”

**Methods:**

We propose a new three‐step algorithm for biological therapy in severe asthma.

**Results:**

Step 1 is to divide subjects into four groups by measuring blood eosinophils and FeNO. Step 2 is to divide the subjects further into six groups by atopy test. In the case of elevated blood eosinophils, normal/elevated FeNO, and atopy, we perform a steroid trial in step 3 in order to decide whether to select anti‐IgE or anti‐IL‐5. The steroid trial is to assess the symptoms of asthma, lung function, blood eosinophils, and FeNO before and after 14 days treatment with 0.5 mg/kg oral prednisolone/day. We judge that cases in which blood eosinophils and FeNO decrease together are not “truely steroid resistance.” In such cases, considering the possibility that allergic type inflammation through adaptive immunity is dominant, anti‐IgE is selected when it is difficult to prevent exacerbations by improving environmental factors. Conversely, we consider that cases in which blood eosinophils and/or FeNO do not decrease, are “truely steroid resistance.” In this case, since there is a possibility that non‐allergic type inflammation due to innate immunity, etc. may remain, anti‐IL‐5, which is expected to be effective for steroid‐resistant eosinophilic inflammation, is selected.

**Conclusions:**

Our three‐step algorithm including the steroid trial may be applicable to companion diagnostics testing for molecularly targeted therapies in severe asthma. Further validation is required to examine the effectiveness of this algorithm.

Asthma is a general, chronic, and heterogeneous disease with different phenotypes. The management of asthma in recent years has become polarized, reflecting the permanence of inhaled corticosteroids (ICS) treatment. Some patients have good control with ICS and/or bronchodilators, while some patients have poor control despite ICS and bronchodilator and/or oral corticosteroid (OCS). Approximately 10% of asthma patients have severe asthma, and they account for 50% of the total asthma‐related costs, which is a major socioeconomic burden.

Molecularly targeted treatment of asthma has made remarkable progress in recent years due to advances in clarifying the molecular pathophysiological mechanism and the development and clinical application of therapeutic drugs corresponding to therapeutic target molecules. Many trials have verified a statistically significant and clinically meaningful reduction in daily maintenance OCS use compared with placebo for patients with severe, uncontrolled OCS‐dependent asthma receiving molecularly targeted treatment 1, 2. Although molecularly targeted treatment shows dramatic efficacy that cannot be experienced with ICS, it does not necessarily show effectiveness in all cases. There are clinical trials where patients were not screened with biomarkers and their effectiveness was not proven 3. From the viewpoint of appropriately personalized medicine and medical economics based on molecular background and biomarkers, it is very important to screen the subjects to be administered molecularly targeted treatment.

Recently, there have been some reports on the therapeutic algorithms for severe asthmatic subjects screened by biomarkers 4, 5. Froidure et al. suggested a decision chart based on current knowledge of the efficacy of add‐on therapies in the most prevalent endo/phenotypes of severe asthma 4. They divided patients into four groups by stratifying according to the blood eosinophils and serum total immunoglobulin E (IgE) (T‐helper [Th] 2‐low asthma, non‐atopic asthma, atopic asthma, and atopic asthma with eosinophilia). Zervas et al. proposed a stepwise therapeutic approach in severe uncontrolled asthmatic subjects 5. They divided the subjects into two groups by stratifying according to the blood eosinophils, sputum eosinophils, atopy, and fraction of exhaled nitric oxide (FeNO) (T2‐high asthma and T2‐low asthma). Moreover, T2‐high asthmatic subjects were divided into three groups: allergic predominance, eosinophilic predominance, and allergic/eosinophilic overlap. These algorithms are roughly similar. However, both algorithms have one problem. In atopic/eosinophilic overlapping patients, there is no effective answer to the question: “which is the optimal choice between Anti‐IgE and anti‐Interleukin 5 (IL‐5)?” In practice, it is known that such overlap subjects are not few. In fact, from a Japanese cohort, about one‐quarter of patients with severe asthma shared allergic/eosinophilic overlap 6.

In terms of how to deal with this problem, we propose a new three‐step algorithm for biological therapy in severe asthma (Fig. 1) that we currently use. Step 1 is to divide subjects into four groups by measuring blood eosinophils and FeNO. Blood eosinophils ≥300 µL^−1^ is defined as elevated blood eosinophils, and FeNO ≥35 ppb is defined as elevated FeNO. Step 2 is to divide the subjects further into six groups by atopy test. Positive specific IgE to at least one allergen was assumed to confirm the presence of atopy. As a result, in the case of normal blood eosinophils, normal FeNO and non‐atopic, we make an alternative approach except for biological therapy. If the patient has normal blood eosinophils, normal/elevated FeNO, and is atopic, anti‐IgE is the treatment of choice. If blood eosinophils are elevated, FeNO is normal/elevated, and the patient is non‐atopic, anti‐IL‐5 is the treatment of choice. In the case of elevated blood eosinophils, normal/elevated FeNO, and atopy, we perform a steroid trial in step 3 in order to decide whether to select anti‐IgE or anti‐IL‐5.

**Figure 1:**
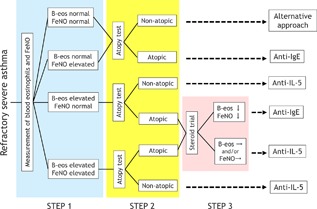
Three‐step algorithm for biological therapy in severe asthma. B‐eos, blood eosinophils; FeNO, fraction of exhaled nitric oxide; IgE, immunoglobulin E; IL‐5, anti‐interleukin 5.

The steroid trial is to assess the symptoms of asthma, lung function, blood eosinophils, and FeNO before and after 14 days treatment with 0.5 mg/kg oral prednisolone/day 7. A recently published paper by ourselves in this journal demonstrates that in severe asthma patients, there was variability in their responses to the steroid trial in FeNO and blood eosinophils, and it was essential to suppress both FeNO and blood eosinophils for the improvement of asthma control 8. One‐half of the subjects showed both reductions in blood eosinophils and FeNO by more than 20%. Here, we judge that cases in which blood eosinophils and FeNO decrease together are not “truely steroid resistance.” In such cases, considering the possibility that allergic type inflammation through adaptive immunity is dominant, anti‐IgE is selected when it is difficult to prevent exacerbations by improving environmental factors. Conversely, we consider that cases in which blood eosinophils and/or FeNO do not decrease, are “truely steroid resistance.” In this case, since there is a possibility that non‐allergic type inflammation due to innate immunity etc. may remain 9, anti‐IL‐5, which is expected to be effective for steroid‐resistant eosinophilic inflammation, is selected.

In fact, we experienced an allergic/eosinophilic overlap asthma case, as shown in Figure 2. We performed the steroid trial, which showed a reduction in blood eosinophils and FeNO and an improvement of symptoms and lung function. Considering the comorbidity of chronic rhinosinusitis with nasal polyps, the possibility of eosinophilic predominance was considered and anti‐IL‐5 could be selected. However, based on the steroid trial results, we selected anti‐IgE. As a result, a significant improvement of biomarkers, symptoms, and lung function was obtained during the anti‐IgE treatment period.

**Figure 2:**
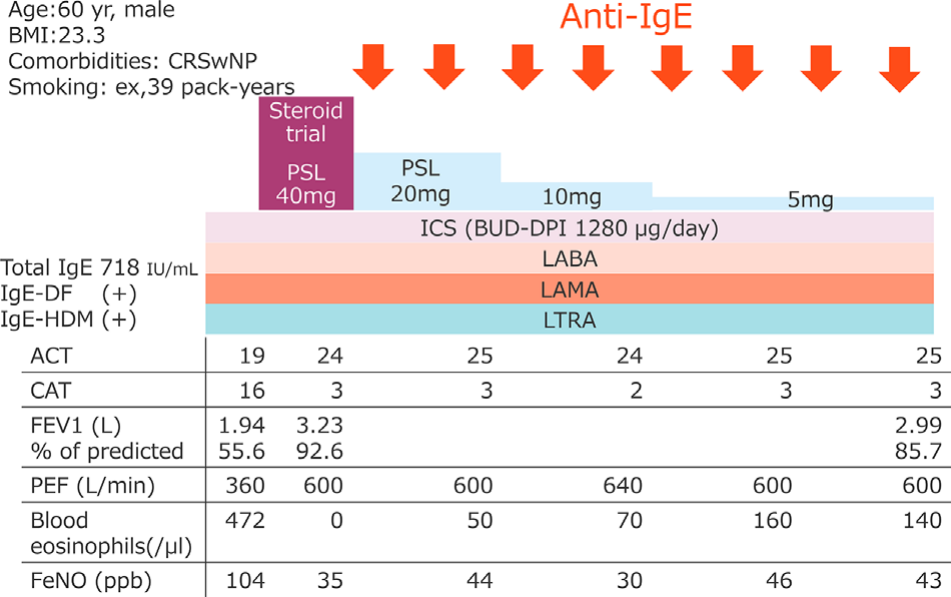
A case of allergic/eosinophilic overlap asthma. CRSwNP: chronic rhinosinusitis with nasal polyps; IgE, immunoglobulin E; DF, dermatophagoides farinae; HDM, house dust mites; PSL, prednisolone; ICS, inhaled corticosteroid; BUD‐DPI, budesonide‐dry powder inhaler; LABA, long‐acting beta‐agonists; LAMA, long‐acting muscarinic antagonists; LTRA, leukotriene receptor antagonist; ACT, Asthma Control Test; CAT, COPD assessment test; FEV1, forced expiratory volume in one second; PEF, peak expiratory flow; FeNO, fraction of exhaled nitric oxide.

There is no direct comparison between anti‐IgE or anti‐IL‐5 in allergic/eosinophilic overlap asthmatics, nor is one planned. Our three‐step algorithm including the steroid trial may be applicable to companion diagnostics testing for molecularly targeted therapies in severe asthma. Further validation is required to examine the effectiveness of this algorithm.

## Ethical Statement

Informed consent was obtained from a patient who presented case.

## Authors’ Contributions

KO and KM designed the study and wrote the manuscript. KO and KM contributed to data collection. KO and KM read and approved the final manuscript.

## Conflict of Interest

The authors declare no conflict of interests.
